# Human platelet activation by *Escherichia coli*: roles for FcγRIIA and integrin αIIbβ3

**DOI:** 10.3109/09537104.2016.1148129

**Published:** 2016-03-30

**Authors:** Callum N. Watson, Steven W. Kerrigan, Dermot Cox, Ian R. Henderson, Steve P. Watson, Mònica Arman

**Affiliations:** ^a^ Institute of Cardiovascular Sciences, College of Medical and Dental Sciences, University of Birmingham, Birmingham, UK; ^b^ Cardiovascular Infection Group, Molecular and Cellular Therapeutics, Royal College of Surgeons in Ireland, Dublin, Ireland; ^c^ Institute of Microbiology and Infection, College of Medical and Dental Sciences, University of Birmingham, Birmingham, UK; ^d^ Centre for Cardiovascular and Metabolic Research, Hull-York Medical School, University of Hull, Hull, UK

**Keywords:** Blood platelets, *Escherichia coli*, Fc gamma receptor IIA, immunity, thrombosis

## Abstract

Gram-negative *Escherichia coli* cause diseases such as sepsis and hemolytic uremic syndrome in which thrombotic disorders can be found. Direct platelet–bacterium interactions might contribute to some of these conditions; however, mechanisms of human platelet activation by *E. coli* leading to thrombus formation are poorly understood. While the IgG receptor FcγRIIA has a key role in platelet response to various Gram-positive species, its role in activation to Gram-negative bacteria is poorly defined. This study aimed to investigate the molecular mechanisms of human platelet activation by *E. coli*, including the potential role of FcγRIIA. Using light-transmission aggregometry, measurements of ATP release and tyrosine-phosphorylation, we investigated the ability of two *E. coli* clinical isolates to activate platelets in plasma, in the presence or absence of specific receptors and signaling inhibitors. Aggregation assays with washed platelets supplemented with IgGs were performed to evaluate the requirement of this plasma component in activation. We found a critical role for the immune receptor FcγRIIA, αIIbβ3, and Src and Syk tyrosine kinases in platelet activation in response to *E. coli*. IgG and αIIbβ3 engagement was required for FcγRIIA activation. Moreover, feedback mediators adenosine 5’-diphosphate (ADP) and thromboxane A_2_ (TxA_2_) were essential for platelet aggregation. These findings suggest that human platelet responses to *E. coli* isolates are similar to those induced by Gram-positive organisms. Our observations support the existence of a central FcγRIIA-mediated pathway by which human platelets respond to both Gram-negative and Gram-positive bacteria.

## Introduction

Platelets have been long known to be activated by bacteria [1]. This is likely to contribute to a balanced immune response [2], but it is also associated with pathological conditions such as infective endocarditis, atherothrombosis, and sepsis [3–5]. In the latter, disseminated microvascular thrombosis has a role in pathophysiology of sepsis and might be mediated through direct platelet–bacterium interactions. Recently, great emphasis has been placed on understanding the molecular mechanisms by which platelets are activated by bacterial cells. Elucidation of such mechanisms would provide opportunities to regulate them during infection. These mechanisms are diverse and include activation by whole bacteria or their released products [1, 6]. Despite multiple bacterial-strain specific molecular interactions, human platelet FcγRIIA is required for activation by a number of different Gram-positive species [7–15] and might contribute to the thrombotic complications found in infective diseases [15]. FcγRIIA is a low-affinity receptor for the constant region of IgGs that recognizes IgG-coated bacteria or their products through avidity. Upon ligand engagement, FcγRIIA signals through Src and Syk tyrosine kinases via a dual YxxL sequence known as an immunoreceptor tyrosine-based activation motif (ITAM) that is present in its cytoplasmic tail [16].

Gram-negative *E. coli* are commensal bacteria of the human and other mammalian gastrointestinal tracts. They rarely cause disease, except in cases of damaged gastrointestinal barriers or immunocompromised hosts. However, pathogenic strains of *E. coli* can cause three general clinical syndromes: enteric/diarrheal disease, urinary tract infections, and sepsis/meningitis [17]. In the latter, *E. coli* strains are the most common Gram-negative bacteria isolated from patients with bacteremia, sepsis, and neonatal meningitis [17–20], causing a major clinical burden and thousands of deaths per year. However, scarce information is available on the molecular interactions between *E. coli* and platelets [21].

The aim of this study was two-fold: to investigate human platelet activation by whole *E. coli* clinical isolates, and to investigate if FcγRIIA mediates platelet activation.

## Materials and methods

### Reagents

All reagents were from described sources [14]. Fibrinogen was from Calbiochem (Merck Millipore, Nottingham, UK) and was depleted of IgGs by incubation with protein A (rec-Protein A-Sepharose 4B Conjugate, Life Technologies [Paisley, UK]).

### Bacterial culture and preparation

*E. coli* strains, CFT073 (isolated from a patient with acute pyelonephritis and bacteremia [22]) and RS218 (isolated from a case of neonatal meningitis [23]), were cultured aerobically at 37°C overnight in an LB broth. Bacteria were washed and adjusted in PBS to an optical density (OD) of 1.6 at a wavelength of 600 nm. Bacteria were used at a 10-fold dilution in aggregation assays unless otherwise indicated.

### Assays of platelet function

Platelet preparation from healthy volunteers was performed as previously described [14]. The study design was approved by the relevant ethics committee (ERN_11-0175). Platelet aggregation was assessed by light transmission in a PAP-8 aggregometer for up to 30 min. Time-matched controls were run alongside. Stimulation by cross-linking of FcγRIIA was performed by preincubation of platelets for 3 min with mAb IV.3 (4 μg/mL) followed by anti-mouse IgG F(ab’)_2_ (30 μg/mL). When indicated, concentrations of both mAb IV.3 and anti-mouse IgG F(ab’)_2_ were doubled or reduced to half. ATP release was assessed at the end of the recording using a luciferin–luciferase based assay [9]. Eptifibatide (9 μM), dasatinib (4 μM), and PRT-060318 (10 μM) were used at supramaximal concentrations.

Cell lysates and protein phosphorylation studies were performed as previously published [14].

### Statistical analysis

Statistical analysis was performed using GraphPad (Prism). Data are presented as mean ± standard deviation (SD), and comparisons between mean values were performed using Student’s *t*-test or ANOVA when multiple samples were compared. *p* < 0.05 (two-tailed) was considered to be significant.

## Results and discussion

### *E. coli* bacteria stimulate αIIbβ3-mediated platelet aggregation via FcγRIIA and Src and Syk tyrosine kinases

**Figure 1.**
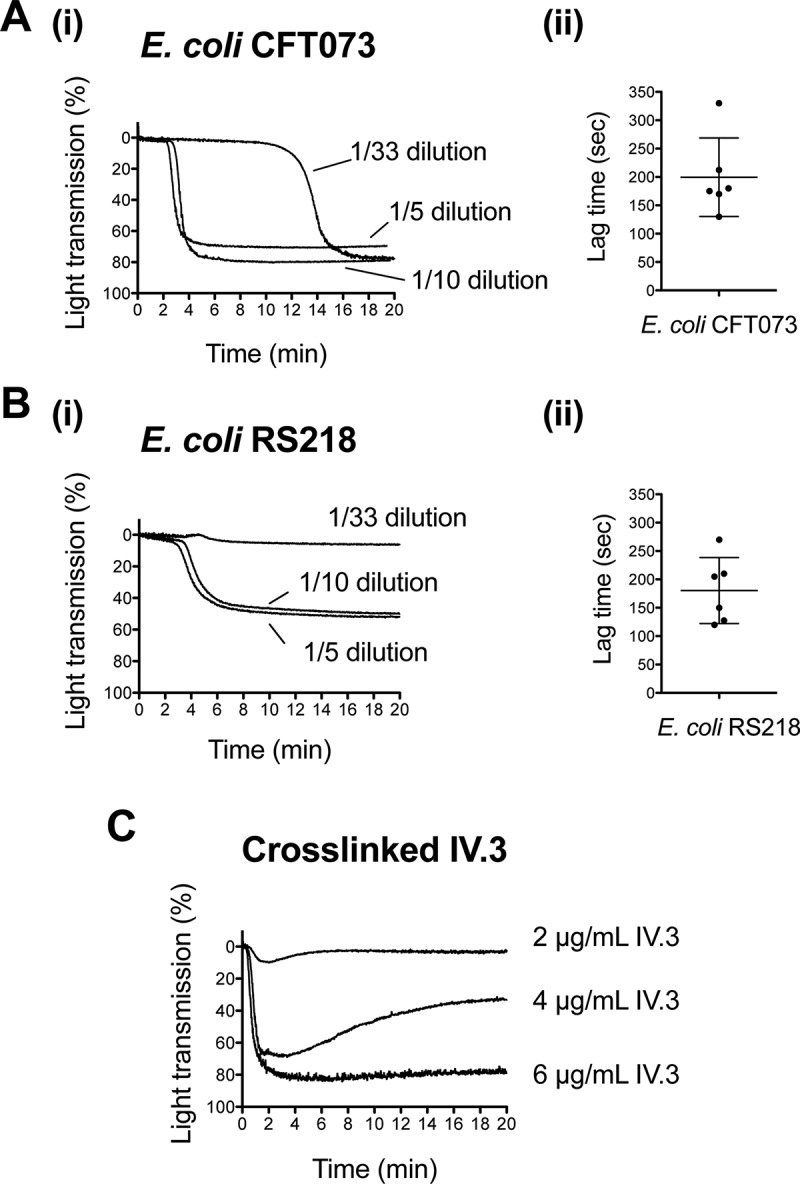
*E. coli* clinical isolates stimulating platelet aggregation in plasma. (A) Effect of *E. coli* CFT073 concentration on the lag time for the onset of platelet aggregation. (A.i) Bacterial suspensions at OD_600nm_ 1.6 were used at different dilutions in platelet aggregation assays as indicated. Platelet-rich plasma (PRP) was 80% of the final volume and was kept constant in all reactions. (A.ii) Lag time for the onset of aggregation was measured in platelets from seven different donors using a 10-fold dilution of the bacterial suspension. Aggregation was observed in six out of seven donors, and lag times are indicated. Experiments were performed on different days (mean ± SD, *n* = 6). (B) Effect of *E. coli* RS218 concentration on the lag time for the onset of platelet aggregation. (B.i) Assays were performed as indicated in A.i. for *E. coli* RS218. (B.ii) Lag time for the onset of aggregation was measured as explained in A.ii. Aggregation was observed in six out of seven donors, and lag times are indicated. Experiments were performed on different days (mean ± SD, *n* = 6). (C) Effect of crosslinked mAb IV.3 concentration on platelet aggregation. In order to crosslink the FcγRIIA receptor, platelets were pre-incubated for 3 min with 2, 4 or 6 μg/mL mAb IV.3 followed by addition of 15, 30, or 45 μg/mL anti-mouse IgG F(ab’)_2_, respectively. Platelet-rich plasma (PRP) was 80% of the final volume and was kept constant in all reactions.

Previous studies have shown a characteristic pattern of platelet activation by Gram-positive bacteria, i.e. they induce “all-or-nothing” aggregation of platelets following a lag time that decreases with increasing concentrations of bacteria [14]. We hypothesized that Gram-negative organisms could trigger platelet aggregation in a similar manner. To test this, we investigated two blood-borne isolates of *E. coli*, CFT073, which was isolated from a patient with urinary tract infection and bacteremia, and RS218, which was isolated from a child with meningitis. We found that both strains induced “all-or-nothing” platelet aggregation in plasma after a lag phase (Figure 1 A.i and B.i). In contrast, as exemplified in Figure 1C for cross-linking of mAb IV.3 to cluster FcγRIIA, most platelet agonists cause rapid activation, which can give rise to partial aggregation when low concentrations of agonist are used. This suggests that bacteria have a unique positive feedback mechanism that gives rise to an “all-or-nothing” response.

The rest of the study was performed with the intermediate bacterial concentration; e.g. bacterial suspensions at OD_600nm_ 1.6 were used at a 10-fold dilution in aggregation assays. Under these experimental conditions and performing the reactions in the presence of plasma, both strains induced platelet aggregation in six out of seven donors tested. For *E. coli* CFT073, the lag time for the onset of aggregation varied from 130 to 330 sec (mean ± SD: 200 sec ± 69, *n* = 6) (Figure 1A.ii). *E. coli* RS218 induced aggregation with lag times ranging from 120 to 270 sec (mean ± SD: 180 sec ± 58, *n* = 6) (Figure 1B.ii).

**Figure 2.**
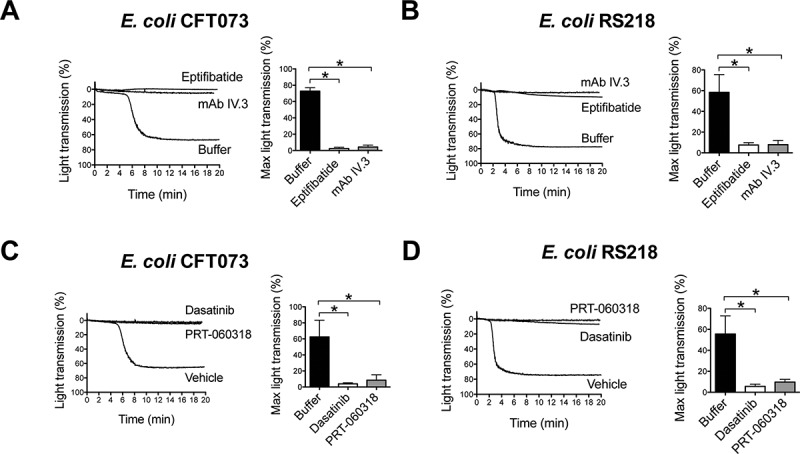
*E. coli*-induced platelet aggregation in plasma depends on FcγRIIA and Src and Syk tyrosine kinases. (A) Effect of αIIbβ3 antagonist, eptifibatide, and anti-FcγRIIA mAb IV.3 on *E. coli* CFT073-induced platelet aggregation. PRP was incubated for 2 min with eptifibatide (9 μM) or 10 min with mAb IV.3 (10 μg/mL) or vehicle prior to addition of bacteria, and platelet aggregation was monitored by light transmission aggregometry. The results on the right-hand side graph are shown as mean ± SD of five independent experiments; *p < 0.05. One representative experiment is shown on the left. (B) Effect of αIIbβ3 antagonist, eptifibatide, and anti-FcγRIIA mAb IV.3 on *E. coli* RS218-induced platelet aggregation. The same experimental conditions as in Figure 2A were used for *E. coli* RS218. The results on the right-hand side graph are shown as mean ± SD of four independent experiments; *p < 0.05. One representative experiment is shown on the left. (C) Effect of the Src-tyrosine kinase inhibitor, dasatinib, and the Syk-tyrosine kinase inhibitor, PRT-060318, in *E. coli* CFT073-induced platelet aggregation in plasma. PRP was incubated for 2 min with dasatinib (4 μM) or PRT-060318 (10 μM) or vehicle prior to addition of bacteria, and platelet aggregation was monitored. The results on the right-hand side graph are shown as mean ± SD of four independent experiments; *p < 0.05. One representative experiment is shown on the left. (D) Effect of the Src-tyrosine kinase inhibitor, dasatinib, and the Syk-tyrosine kinase inhibitor, PRT-060318, in *E. coli* RS218-induced platelet aggregation in plasma. The same experimental conditions as in Figure 2C were used for *E. coli* RS218. The results on the right-hand side graph are shown as mean ± SD of four independent experiments; *p < 0.05. One representative experiment is shown on the left.

As shown in Figure 2A and B, platelet aggregation to *E. coli* CFT073 and RS218 was blocked in the presence of the αIIbβ3 antagonist eptifibatide, which confirmed that the change in light transmission was due to αIIbβ3-mediated platelet–platelet binding rather than passive agglutination.

To analyze whether FcγRIIA and its signaling pathway components, Src and Syk tyrosine kinases, have a role in *E. coli* induced platelet aggregation, specific inhibitors were employed. Aggregation induced by *E. coli* CFT073 or RS218 strains was abolished when platelets were pre-incubated with either mAb IV.3 (FcγRIIA inhibitor, Figure 2A and 2B), dasatinib (Src inhibitor, Figure 2C and 2D), or PRT-060318 (Syk inhibitor, Figure 2C and 2D) in the presence of plasma.

### Platelet activation by *E. coli* requires the interplay between αIIbβ3 and FcγRIIA

**Figure 3.**
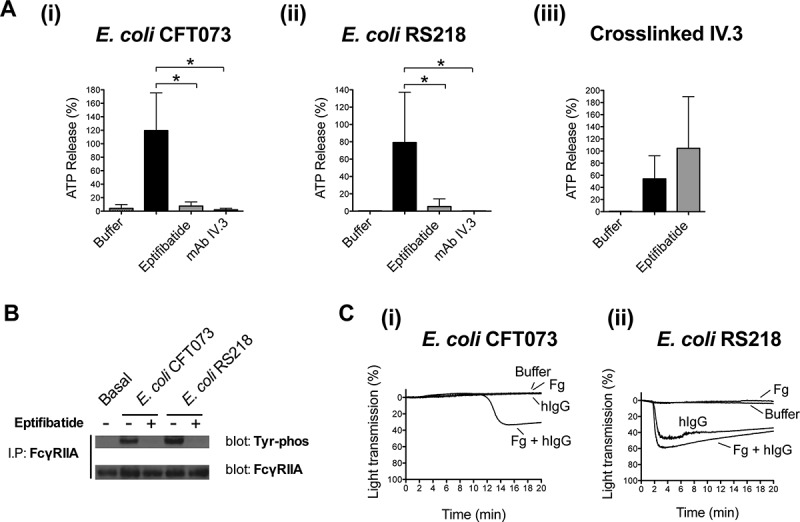
Platelet activation by *E. coli* requires the interplay between αIIbβ3 and FcγRIIA. (A) αIIbβ3 and FcγRIIA mediate *E. coli* induced platelet secretion. Platelet-rich plasma was incubated for 2 min with eptifibatide (9 μM) or 10 min with mAb IV.3 (10 μg/mL) or vehicle prior to addition of bacteria, and platelet aggregation was monitored by light transmission aggregometry. Reactions were also performed by crosslinking FcγRIIA receptor. Platelets were pre-incubated for 3 min with 4 μg/mL monoclonal antibody (mAb) IV.3 followed by addition of 30 μg/mL anti-mouse IgG F(ab’)_2_. Supernatants were collected at time of full aggregation, or a parallel time point in the case of inhibition. Supernatants were analyzed for ATP release by luciferin–luciferase assay. ATP levels released by TRAP (100 μM) stimulated platelets were used to normalize data. The results are shown as mean ± SD of five independent experiments for *E. coli* CFT073 and RS218 and three independent experiments for crosslinked mAb IV.3; *p < 0.05. (B) *E. coli* induces tyrosine phosphorylation of FcγRIIA that is dependent on αIIbβ3. Aggregation reactions were performed in plasma in the presence or absence of eptifibatide (9 μM) and cell lysates collected at time of full aggregation, or equivalent times in the case of eptifibatide-treated samples where aggregation was inhibited. Immunoprecipitations for FcγRIIA were performed and tyrosine-phosphorylation detected by Western blot. Representative results of three independent experiments are shown. (C) hIgGs reconstitute aggregation to *E. coli* in washed platelets. Aggregation reactions to *E. coli* CFT073 and *E. coli* RS218 were performed in washed platelets in the presence or absence of IgG-depleted fibrinogen (1 mg/mL) and pooled human IgGs from healthy donors (hIgG) (0.1 mg/mL). Three independent experiments were performed per strain using different platelet donors. One representative experiment is shown for *E. coli* RS218. For *E. coli* CFT073, platelet aggregation was restored in two out of three platelet donors when supplementing the reactions with fibrinogen plus hIgG, and one representative experiment is shown.

A key role for FcγRIIA in platelet activation by *E. coli* is further supported by the observation that dense granule secretion was inhibited by mAb IV.3 (Figure 3A.i and A.ii) and that FcγRIIA became phosphorylated in response to both *E. coli* CFT073 and RS218 strains (Figure 3B). Furthermore, while washed platelets were not able to support bacteria-mediated aggregation, aggregation to *E. coli* RS218 was restored in the presence of human IgGs (e.g. pooled human IgGs purified from healthy donors) alone or with fibrinogen (Figure 3C.ii). Simultaneous addition of human IgGs and fibrinogen was necessary for *E. coli* CFT073 to induce aggregation in washed platelets (Figure 3C.i). These observations suggest that the initiating event in activation is engagement of FcγRIIA by plasma IgG bound to bacteria.

Research on Gram-positive bacteria has shown that platelet activation is often the result of multiple bacterium–platelet molecular interactions. These include the combination of bacterial strain-specific molecular interactions and shared IgG-FcγRIIA mediated signaling events [6, 14]. Among the former, strain-specific streptococci and staphylococci proteins are found that bind directly or indirectly (e.g. via fibrinogen) to platelet surface receptors such as αIIbβ3 or GPIb [6, 24]. Our results suggest that the mechanisms of platelet activation by *E. coli* might also have a strain-dependent component. However, future work is necessary to characterize the exact molecular interactions between platelets and these two *E. coli* strains, including the identification of potential bacterial components binding (directly or indirectly) to platelet surface receptors.

Previous studies demonstrated an unexpected role for αIIbβ3 in controlling platelet dense granule secretion and FcγRIIA phosphorylation in response to a wide range of Gram-positive bacteria [13, 14]. We analyzed whether the same pattern of regulation could take place for *E. coli*. Indeed, *E. coli* CFT073 and RS218-induced dense granule secretion was inhibited by eptifibatide (Figure 3A.i and 3A.ii) demonstrating that secretion is dependent on αIIbβ3 engagement. In contrast, secretion induced by cross-linked mAb IV.3 was not decreased by eptifibatide (Figure 3A.iii). Moreover, bacteria-induced tyrosine phosphorylation of FcγRIIA was also dependent on αIIbβ3 engagement as observed by the inhibition of phosphorylation in the presence of eptifibatide (Figure 3B).

The inability to detect secretion and FcγRIIA tyrosine phosphorylation in the absence of αIIbβ3 engagement might reflect the weak nature of the pathway initiated after FcγRIIA engagement by IgG-coated bacteria in the absence of feedback signals. The mechanism by which initial αIIbβ3 engagement takes place is, however, unclear. Strain-specific events mediating αIIbβ3 activation are thought to exist for Gram-positive bacteria. For most cases, it is likely that αIIbβ3 inside-out activation is achieved by FcγRIIA signaling, as well as by signaling from other strain-specific bacterial ligand–platelet receptor pairs. However, some bacteria such as *Streptococcus gordonii* DL1 and *Staphylococcus aureus* Newman can bind directly or indirectly (e.g. via fibrinogen) to αIIbβ3, which could facilitate αIIbβ3 activation [1, 10, 25–27]. Further investigations are necessary to characterize the mechanisms that lead to αIIbβ3 activation by *E. coli* CFT073 and RS218, including the identification of potential *E. coli* ligands binding to αIIbβ3.

### Platelet activation by *E. coli* is also dependent on secondary mediators ADP and TxA_2_

**Figure 4.**
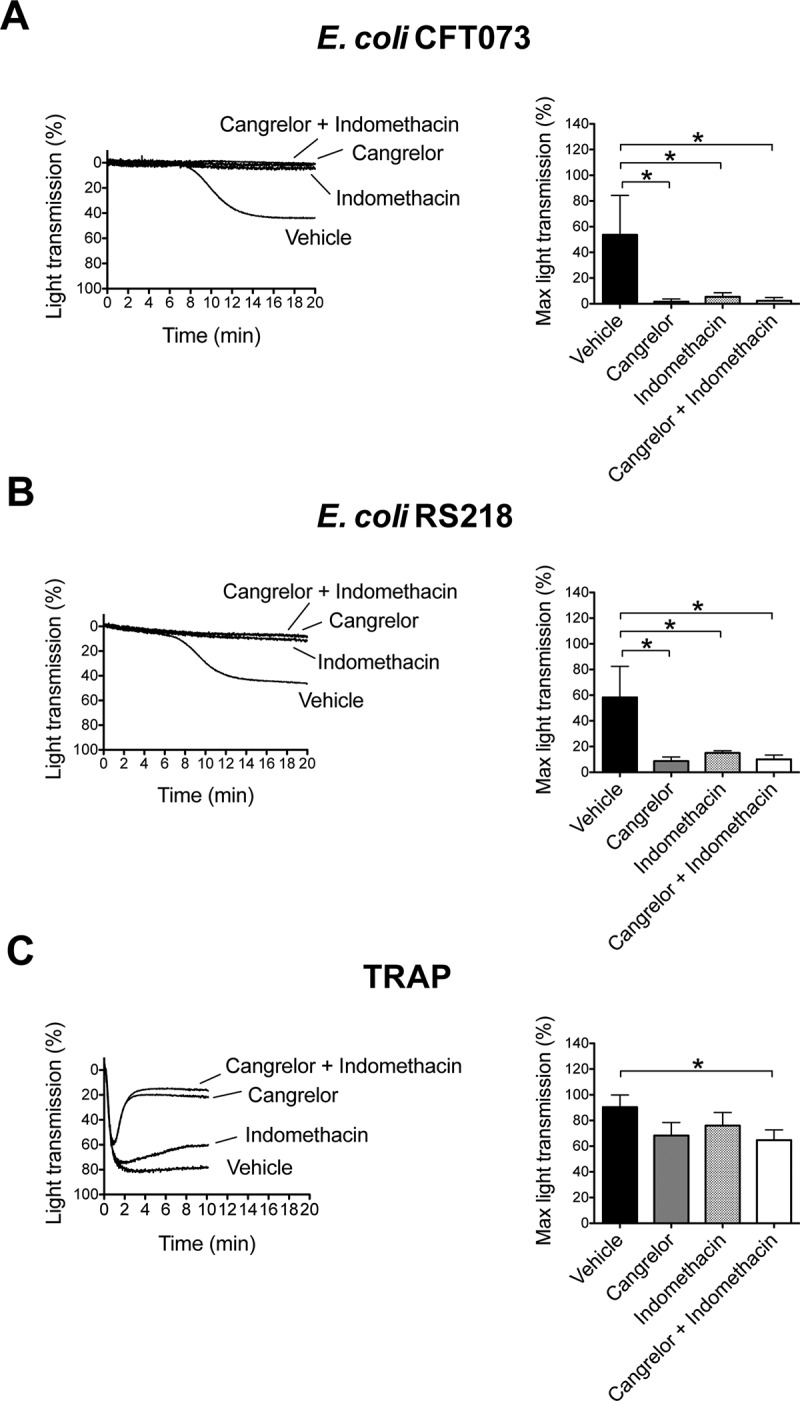
Platelet activation by *E. coli* depends on secondary mediators ADP and T_x_A_2_. Platelet-rich plasma was incubated for 2 min with the cyclooxygendase inhibitor indomethacin (10 μM), ADP-receptor P2Y_12_ inhibitor Cangrelor (1 μM), or vehicle (DMSO) prior to addition of *E. coli* CFT073 (A), *E. coli* RS218 (B), or 50 μM TRAP (C), and platelet aggregation was monitored. The results are shown as mean ± SD of three independent experiments; *p < 0.05.

Platelet activation is reinforced by secondary mediators, which include release of stored ADP from dense granules and *de novo* synthesis of TxA_2_. Positive feedback mechanisms driven by secondary mediators are normally required for full and/or sustained platelet aggregation to low concentrations of agonists. This can be seen in platelets stimulated with an intermediate concentration of thrombin-related peptide (TRAP). Pre-treatment of platelets with the ADP-receptor P2Y_12_ antagonist, Cangrelor, and/or with cyclooxygenase inhibitor, indomethacin, did not affect the initial TRAP-induced aggregation but was followed by slow deaggregation that was not seen in controls (Figure 4C).

In contrast, secondary mediators are key for platelet activation to *Staphylococcus* and *Streptococcus* strains [14]. Here, inhibitors were used to investigate a role for the two feedback agonists in *E. coli*-induced platelet aggregation. Activation in response to *E. coli* CFT073 and RS218 was completely abrogated in the presence of either Cangrelor or indomethacin, or by the combination of both (Fig 4A and 4B). Thus, platelet activation by *E. coli* bacteria is also dependent on ADP and TxA_2_.

Altogether, these data suggest that the combination of FcγRIIA activation upon recognition of IgG-coated bacteria plus αIIbβ3 engagement (e.g. either through binding to bacteria or as a result of inside-out platelet signaling events as discussed before) results in a weak signal leading to release of ADP and TxA_2_. At this stage, feedback mechanisms are key in order to achieve full activation. Furthermore, ADP and TxA_2_ signal to neighboring (bacteria-free) platelets and induce αIIbβ3 inside-out activation and consequent platelet–platelet aggregation.

Interestingly, FcγRIIA has also been shown to function as an adaptor protein amplifying αIIbβ3 signaling independent of extracellular IgG engagement [28, 29]. This suggests that FcγRIIA and αIIbβ3 could support both initial platelet–bacterium interaction and subsequent platelet–platelet aggregation by means of cooperative integrin/immunoreceptor tyrosine-based activation motif signaling.

In summary, in this study, we provide evidence that *E. coli* induces activation of platelets through the same shared pathway described for various Gram-positive *Staphylococcus* and *Streptococcus* species [14]. This pathway involves IgG-dependent FcγRIIA activation of Src and Syk kinases, and is reinforced by αIIbβ3 engagement and secondary mediators. Despite the fact that FcγRIIA-mediated aggregation was previously observed for Gram-negative *Helicobacter pylori* [30] and *Porphyromonas gingivalis* [31], the signaling pathway and role of αIIbβ3 in activation has been only evaluated in few Gram-positive species [10, 13, 14]. The demonstration of a common mode of platelet activation to Gram-positive and Gram-negative species further identifies FcγRIIA as a candidate receptor for prevention of bacteria-mediated platelet activation in thrombosis and related disorders.

We found that platelets from one out of seven donors did not respond to either *E. coli* CFT073 or *E. coli* RS218. Donor variation in bacteria-induced platelet aggregation is common and has been reported before [32, 33]. Future studies using a larger number of donors will be required in order to evaluate the potential effect of plasma IgG levels and FcγRIIA polymorphisms and/or surface expression levels on human platelet aggregation in response to *E. coli*. However, previous studies have not found a clear correlation between donor response and the above parameters in the case of Gram-positive bacteria [32, 33] and, for *Streptococcus sanguinis*, IgG levels can only partially account for donor variability [33].

As previously mentioned, individual bacterial strains can mediate platelet–bacterium interactions by multiple receptor–ligand pairs [1, 6], each one having a different contribution to the adhesion and/or platelet activation steps. Although we have found that FcγRIIA has a key role in human platelet activation by *E. coli* CFT073 and RS218, other platelet receptors might be simultaneously interacting with these strains, and this should be investigated in future studies. Furthermore, the bacterial components that are being targeted by IgGs-FcγRIIA remain to be identified. Previous literature shows that *E. coli* secreted Shiga-toxin, which is a virulent factor associated with hemolytic uremic syndrome [34], induces platelet activation [35]. And this might contribute to the formation of platelet thrombi in kidney glomerular capillaries, small arterioles, and arteries [34]. Additionally, human platelets bind lipopolysaccharide (LPS) from enterohemorrhagic *E. coli* via toll-like receptor (TLR) 4 and CD62, leading to cell activation [36]. TLR4-mediated platelet cytokine secretion has been described in response to *E. coli* LPS [37]. While *E. coli* CFT073 and RS218 do not produce Shiga-toxin, a role for *E. coli* CFT073 and RS218 cell wall LPS in platelet activation cannot be discarded, either in relation to FcγRIIA-mediated events (via IgG) or independently of the IgG receptor. As a first approach to test the role of TLR4 in platelet activation in response to our *E. coli* strains, we used an inhibitory anti-TLR4 antibody, HTA125, and found that it had no effect on platelet aggregation, and did not prolong the lag time response to either *E. coli* CFT073 or *E. coli* RS218 using two different donors (data not provided). This suggests that TLR4 is not essential for platelet activation by the bacterial strains tested. However, it is still possible that *E. coli* LPS-IgG immune complexes are formed that can engage platelet FcγRIIA directly. In any case, the exact role of *E. coli* CFT073 and RS218 LPS and platelet TLR4 in mediating platelet activation still needs to be deciphered.

Interestingly, one previous study showed that FcγRIIA was required for platelet-mediated killing of IgG-opsonized *E. coli* K12 [38]. This suggests that platelet activation by bacteria might have different outcomes depending on the overall scenario: while unbalanced thrombi formation might have detrimental effects in cases such as HUS or sepsis, platelet activation by *E. coli* coated with IgG found in sera from healthy individuals (i.e. such ones used in this study) might contribute to host defense. Future investigations are necessary to further decipher the molecular interactions between *E. coli* and platelets, including potential synergism between IgG-FcγRIIA and LPS-TLR mediated pathways, and their role in homeostasis or disease. Furthermore, in view of the present study, care should be taken when using animal models for the study of platelet function during *E. coli* infections as FcγRIIA is not found in mice.
